# 5'-Serial Analysis of Gene Expression studies reveal a transcriptomic switch during fruiting body development in *Coprinopsis cinerea*

**DOI:** 10.1186/1471-2164-14-195

**Published:** 2013-03-20

**Authors:** Chi Keung Cheng, Chun Hang Au, Sarah K Wilke, Jason E Stajich, Miriam E Zolan, Patricia J Pukkila, Hoi Shan Kwan

**Affiliations:** 1 Food Research Centre and Food and Nutrition Sciences Programme, School of Life Sciences, Faculty of Science, The Chinese University of Hong Kong, Shatin, S.A.R., Hong Kong; 2 Department of Biology, University of North Carolina at Chapel Hill, Chapel Hill, North Carolina, USA; 3 Department of Plant Pathology and Microbiology, University of California-Riverside, Riverside, United States of America; 4 Department of Biology, Indiana University, Bloomington, Indiana, United States of America

**Keywords:** *Coprinopsis cinerea*, SAGE, Transcriptome, Microarray, Transcription start site, Fruiting body, Mycelium, Primordium

## Abstract

**Background:**

The transition from the vegetative mycelium to the primordium during fruiting body development is the most complex and critical developmental event in the life cycle of many basidiomycete fungi. Understanding the molecular mechanisms underlying this process has long been a goal of research on basidiomycetes. Large scale assessment of the expressed transcriptomes of these developmental stages will facilitate the generation of a more comprehensive picture of the mushroom fruiting process. In this study, we coupled 5'-Serial Analysis of Gene Expression (5'-SAGE) to high-throughput pyrosequencing from 454 Life Sciences to analyze the transcriptomes and identify up-regulated genes among vegetative mycelium (Myc) and stage 1 primordium (S1-Pri) of *Coprinopsis cinerea* during fruiting body development.

**Results:**

We evaluated the expression of >3,000 genes in the two respective growth stages and discovered that almost one-third of these genes were preferentially expressed in either stage. This identified a significant turnover of the transcriptome during the course of fruiting body development. Additionally, we annotated more than 79,000 transcription start sites (TSSs) based on the transcriptomes of the mycelium and stage 1 primoridum stages. Patterns of enrichment based on gene annotations from the GO and KEGG databases indicated that various structural and functional protein families were uniquely employed in either stage and that during primordial growth, cellular metabolism is highly up-regulated. Various signaling pathways such as the cAMP-PKA, MAPK and TOR pathways were also identified as up-regulated, consistent with the model that sensing of nutrient levels and the environment are important in this developmental transition. More than 100 up-regulated genes were also found to be unique to mushroom forming basidiomycetes, highlighting the novelty of fruiting body development in the fungal kingdom.

**Conclusions:**

We implicated a wealth of new candidate genes important to early stages of mushroom fruiting development, though their precise molecular functions and biological roles are not yet fully known. This study serves to advance our understanding of the molecular mechanisms of fruiting body development in the model mushroom *C. cinerea*.

## Background

The mystery of mushroom formation has fostered a deep curiosity for understanding the molecular mechanisms underlying fruiting body initiation and development in basidiomycetes and a goal of mycological research community. *Coprinopsis cinerea* (previously known as *Coprinus cinereus*), the inky cap, is a model mushroom commonly employed to study developmental processes in agaricomycetous fungi [1]. In addition to studies related to mating types, enzyme production, and genome manipulation, much emphasis has been put on the fruiting process, particularly on the later stages including synchronous karyogamy and meiosis. This process is the most complex, yet rapid, of the developmental events in the life cycle of *C. cinereus*. When nutrients are depleted, the relatively loose mesh of undifferentiated mycelium undergoes a drastic change to form a compact multihyphal structure with many different cell types holding each other through hyphal-hyphal interactions, known as the fruiting body [2]. The fruiting process can be divided into six main developmental stages: hyphal knot, initial, stage 1 primordium, stage 2 primordium, immature fruiting body and mature fruiting body [1]. Besides nutritional constraints, the normal day-night rhythm cycle is also critical for the transition of one stage to another [3,4]. Under ideal conditions, the whole process takes only a few days following the first sign of fruiting [5].

The advancement from mycelium (Myc) into stage 1 primordium (S1-Pri) is hypothesized to show the most significant transcriptomic changes as it represents development from an undifferentiated structure to a well-organized multihyphal structure at a later stage of fruiting body formation. A number of genes have been previously characterized to be involved during this transition. These include the oligosaccharide-binding galectins *cgl1* and *cgl2* for potential but yet unproven mediation of hyphal interactions [6,7]; a homolog to the bacterial cyclopropane fatty acid synthase for generating stress signals as a result of membrane alteration [8]; blue light photoreceptors *dst1* and *dst2* for photomorphogenesis [3,4]; a *Ustilago maydis* adaptor protein Ubc2 homolog for filamentous growth, pheromone response and virulence [9] and a number of metabolic enzymes such as adenylate cyclase, phenol oxidase and glycogen phosphorylase [1]. Nevertheless, the lack of large scale assessment of gene expression hinders the generation of a more comprehensive picture of the fruiting process. In order to better understand the molecular basis underlying this transition, it is desirable to identify the genes differentially expressed among these two developmental stages through comparing the respective transcriptomes. Such studies are especially feasible in *C. cinerea* because of the recent release of its 37.5 Mb genomic sequence and predicted gene set consisting of 13,342 protein-coding genes [10].

Serial Analysis of Gene Expression (SAGE), invented in 1995, is a digital expression tag profiling technology for high throughput genomic-level evaluation of transcriptomes [11]. Over the past 17 years, SAGE has become a well-recognized tool and has been extensively used in human, animal, yeast, plant and fungal studies [12]. The advancement of next-generation DNA sequencing technologies in recent years has enabled SAGE analysis to attain higher throughput, sensitivity and cost-effectiveness compared to the original cloning-based and Sanger sequencing approaches [13-15]. In addition, the SAGE protocol was improved based on a combination of 5^′^ RACE and SAGE, known as 5^′^-SAGE, which extracts the first 15–17 bp of each mRNA transcript [16,17]. This allows simultaneous characterization of the transcriptome and transcription start site (TSS). The TSS data are invaluable for identification and analyses of promoter regions and *cis*-regulatory elements that contribute to better understanding of higher-order regulatory mechanisms [18]. The >100 bp sequence reads available with 454 Life Sciences (GS20) sequence technology [19] is well-suited for the length of 5^′^-SAGE ditags. The short length, but more numerous sequences from the 454 approach allows the tedious steps of concatenation and colony picking in the original SAGE procedures to be bypassed.

Here, we report a high throughput analysis of the two 5^′^-SAGE libraries, supplemented by oligomicroarray data, for comprehensive assessment of the transcriptomes of *C. cinereus* at the Myc and S1-Pri stages. A wealth of novel candidate genes and molecular mechanisms related to fruiting body initiation and development are identified from the comparison of the gene expression profiles at these stages. The genes and pathways identified will serve as an important platform for future studies in developmental biology of basidiomycetes.

## Results and discussion

### Sequencing of 5^′^-SAGE ditags and separation into tags

The 5^′^-SAGE libraries were constructed from each Myc and S1-Pri mRNA preparations and we obtained a total of 198,809 valid ditags from both developmental stages. At an average read length of 95.5 bases, the sequences accounted for 94.9% of the GS20 sequencer throughput. After tag extraction and quality filtering, we obtained a total of 253,415 valid tags, with 107,046 from the Myc stage and 146,369 from the S1-Pri stage.

### Tag to gene assignment

All the Myc and S1-Pri tags were assigned to the *C. cinerea* genome assembly [10] by in-house Perl scripts and no mismatches were allowed. We found approximately 80% of the 107,046 Myc tags and the 146,369 S1-Pri tags matched perfectly to the genome with no mismatches (Table 1). Such percentage is comparable to previous studies using similar techniques [16,17,20]. Meanwhile, an average of 7.2% of tags matched to multiple positions and 11.3% could not be mapped to the genome (Table 1). Most of the multiple mapping tags (>90%) were perfectly aligned to 2–4 genomic positions of non-homologous genes but were discarded from further analysis due to the impossibility of confidently assigning them to unique genome positions. The tags which failed to match the genome were likely the result of unique sequence in the strain sequence discrepancies between the reference monokaryotic strain (Okayama-7 #130) and the dikaryon used in this study. In addition, as suggested by Keime *et al*. [21], a smaller proportion could result from tags spanning splice junction of two exons. The tags mapped to the *C. cinerea* genome and predicted gene models can be retrieved in the link http://kwanlab.bio.cuhk.edu.hk/cgi-bin/gb2/gbrowse/cc.

**Table 1:** **Summary of tag extraction and *C. cinerea *genome mapping**

	**Myc 5**^**′**^**-SAGE**	**S1-Pri 5**^**′**^**-SAGE**
**Total valid tags**	107046	146369
**Genome mapping**		
- Unique match to genome	88053 (82.3%)	118257 (80.8%)
- Multiple matches to genome	7622 (7.1%)	10685 (7.3%)
- 2–4 matches	7065 (6.6%)	9953 (6.8%)
- 5–10 matches	428 (0.4%)	586 (0.4%)
- >11 matches	138 (0.1%)	146 (0.1%)
- No match to genome	11371 (10.6%)	17427 (11.9%)

**Figure 1:**
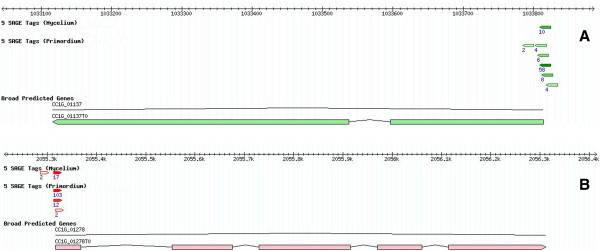
**Alternative transcription start sites (TSSs).** Mapping of 5^′^-SAGE tags to **(A)** Gene CC1G_01137 encoding dimethylribityllumazine synthase and **(B)** Gene CC1G_01278 encoding proteasome subunit beta type 6. The tags demonstrated an array of transcription start sites (TSSs) in the expressed genes with moderate to high expression levels and that there are one or two more preferred TSSs.

**Figure 2:**
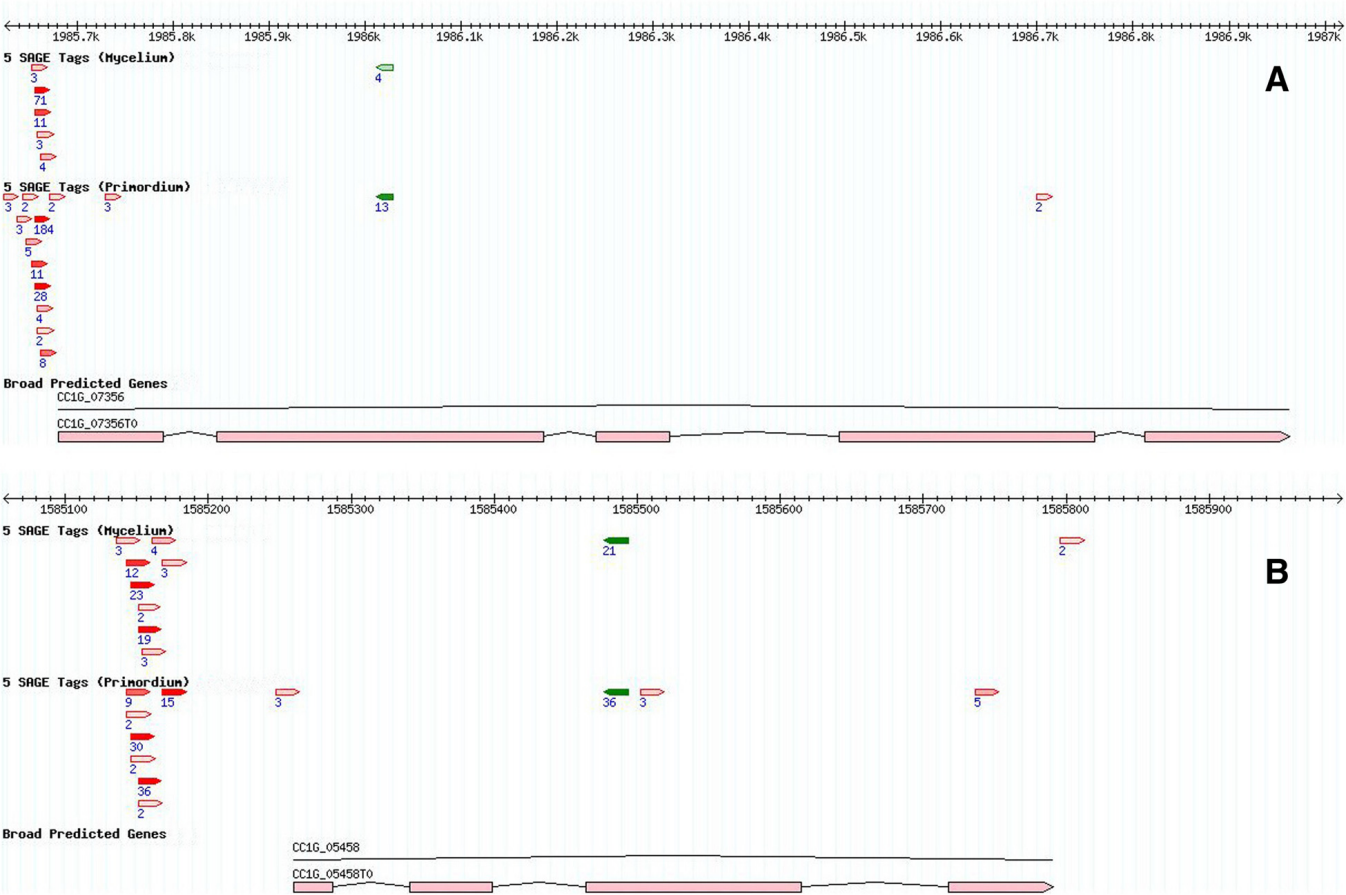
**Anti-sense transcription in annotated coding exons.** Mapping of 5^′^-SAGE tags in anti-sense orientation (green tags) to **(A)** Gene CC1G_07356 encoding G-protein beta subunit and **(B)** Gene CC1G_05458 encoding histone H4. The tags were mapped to the annotated coding exons and they were identical in sequences in both the Myc and S1-Pri libraries, suggesting that they did not occur spuriously and may play potential regulatory roles in expression of the sense transcripts.

Most transcription start sites (TSSs) were located within 200 bp upstream of the translation start codon, and more than 90% of the tags with more than 2 occurrences were within 500 bp upstream of an annotated gene (data not shown). Combining these observations and those from previous reports [22-24], we adopted a length of 500 bp for the putative 5^′^-untranslated region (5^′^-UTR) and 500 bp for the 3^′^-UTR. We observed that 56.5% of the Myc tags and 52.4% of S1-Pri tags mapped to the 5^′^-UTR, 15.0% and 17.1% to the annotated coding region, and 3.3% and 2.6% to the 3^′^-UTR respectively (Table 2). Considering the tags mapping to the 5^′^-UTR, we annotated more than 34,000 Myc and more than 45,000 S1-Pri specific TSSs (79,000 total) of more than 3,000 genes. Most of these start sites were arranged in an array manner (Figure 1) and such arrangement agrees with the observations in many other organisms [17,20,25,26]. We believe the 5^′^-SAGE tags represent genuine transcription start sites and support the accuracy of the computer-predicted gene models. Investigation of the most highly expressed genes revealed that most have more than one TSS with one or two more preferred TSSs (Figure 1). Intriguingly, noticeable proportions (14.3% in Myc and 17.4% in S1-Pri) of tags were mapped in antisense direction to annotated genes, especially to the 5^′^-UTR and coding region (Table 2). Some of these tags could be identified in both the Myc and S1-Pri libraries, implying that they did not occur spuriously (Figure 2). We speculate that they may represent TSS of non-coding RNAs (ncRNAs) with regulatory functions that control gene expression [27-29]. However, further analysis of these non-coding RNAs is beyond the scope of the present study.

**Table 2:** **Gene-associated positions of tags mapped to the *C. cinerea *genome**

	**Myc 5**^**′**^**-SAGE**	**S1-Pri 5**^**′**^**-SAGE**
Putative 5^′^-UTR (−500, -1) ^a^	49718 (56.5%)	61964 (52.4%)
Putative 5^′^-UTR (Anti-sense) ^b^	4363 (5.0%)	8826 (7.5%)
Coding region (sense)	13201 (15.0%)	20195 (17.1%)
Coding region (Anti-sense) ^b^	6030 (6.8%)	8902 (7.5%)
Putative 3^′^-UTR (+1, +500) ^a^	2867 (3.3%)	3031 (2.6%)
Putative 3^′^-UTR (Anti-sense) ^b^	2231 (2.5%)	2889 (2.4%)
Unclassified ^c^	9643 (10.9%)	12450 (10.5%)
**Total**	**88053** (100%)	**118257** (100%)

^a^ The (−500, -1) refers to position upstream of the annotated ATG start codon, while (+1, +500) refers to position downstream of the STOP codon.

^b^ ‘Anti-sense’ means that the tag is in opposite orientation of the putative 5^′^/3^′^-UTR, or annotated coding region.

^c^ ‘Unclassified’ means that the tags could not be assigned to any annotated gene.

### Profiling and functional annotation of the differentially expressed genes (DEGs)

To evaluate the expression level of genes, we only considered the tags mapping to the 5^′^-UTR regions of individual genes. We evaluated the expression level of a total of 3,270 genes (Additional file 1), of which 1,911 and 2,732 were expressed in Myc and S1-Pri stages respectively (Table 3). This represented approximately one-fourth of the 13,342 predicted protein-coding genes (Broad Institute, assembly version 3) [30] in the *C. cinerea* genome. Gene expression of these genes was tested with a Fisher’s Exact Test comparing observed tag counts out of the entire and an unadjusted *p*-value of 0.05. 324 genes were found to be preferentially expressed in Myc (Table 4, Additional file 2) while 716 genes were up-regulated in S1-Pri (Table 5, Additional file 3). This indicates that almost one-third of the assessed genes were up-regulated in either stage, implying a significant turnover of the transcriptome during the transition from mycelium to primordium. Most of the DEGs had shown more than 3-fold up-regulation in either Myc (91%) or S1-Pri (75%) stages (Table 3).

**Table 3:** Statistics of the Myc and S1-Pri differentially expressed genes (DEGs)

	**Myc 5**^**′**^**-SAGE**	**S1-Pri 5**^′^**-SAGE**
No. of genes with detected expression	1911	2732
No. of differentially expressed genes (DEGs)^a^	324 (17.0%)	716 (26.2%)
No of DEGs		
with homolog identified in BLASTX^b^	157 (48.5%)	500 (69.8%)
with >3-fold up-regulation	295 (91.0%)	538 (75.1%)
with GO term	146 (45.1%)	530 (74.0%)
with KEGG ortholog	56 (17.3%)	270 (37.7%)
unique to fruiting body-forming	22 (6.8%)	30 (4.2%)
basidiomycetes^c^		
unique to *C. cinerea*	41 (12.7%)	22 (3.1%)
No. of enriched protein domains^d^	118	115

^a^ p<0.05 in Fisher’s exact test.

^b^ e<10^-5^ in BLASTX homology search.

^c^ Homologs identified in at least two fruiting body-forming basidiomycetes.

^d^ p<0.05 in Fisher’s exact test.

**Table 4:** List of top 15 differentially expressed genes in the Myc stage

**Gene ID**	**Myc**^**a**^	**S1-Pri**^**a**^	**Log**_**2**_**ratio**^**b**^	**BLASTX homolog [Organism]**	**Accession ID**	**e-value**	**KO term**^**c**^	**GO term**^**d**^
CC1G_02184T0	879.2	0.0	9.78	CoH1 [Coprinopsis cinerea]	CAA71652	2.85E-30	N/A	N/A
CC1G_07511T0	752.1	0.0	9.55	hemerythrin HHE cation binding domain protein [Neosartorya fischeri NRRL 181]	XP_001261781	3.71E-13	N/A	N/A
CC1G_08199T0	516.0	0.0	9.01				N/A	N/A
CC1G_08735T0	482.7	0.0	8.92				N/A	N/A
CC1G_11444T0	460.0	0.0	8.85				N/A	GO:0005634
CC1G_12265T0	970.0	2.1	8.83				N/A	GO:0005811 GO:0004252
CC1G_02183T0	729.4	2.1	8.42	CoH1 [Coprinopsis cinerea]	CAA71652	6.28E-29	N/A	N/A
CC1G_13813T0	293.6	0.0	8.20				N/A	N/A
CC1G_07100T0	272.4	0.0	8.09				N/A	N/A
CC1G_10342T0	254.2	0.0	7.99				N/A	N/A
CC1G_11525T0	236.1	0.0	7.88	putative phosphatidic acid phosphatase [Pleurotus sp. &apos;Florida&apos;]	CAD10795	3.17E-47	N/A	GO:0005887 GO:0008195 GO:0006644
CC1G_00675T0	230.0	0.0	7.85				N/A	N/A
CC1G_07531T0	151.3	0.0	7.24				N/A	N/A
CC1G_04294T0	149.8	0.0	7.23				N/A	N/A
CC1G_12264T0	139.2	0.0	7.12				N/A	N/A

^a^ Relative expression level expressed as tags per 100,000.

^b^ Log_2_ of the expression ratio of Myc/S1 Pri.

^c^ KEGG orthology term.

^d^ Gene Ontology term.

**Table 5:** List of top 15 differentially expressed genes in the S1 Pri stage

**Gene ID**	**Myc**^**a**^	**S1-Pri**^**a**^	**Log**_**2**_**ratio**^**b**^	**BLASTX homolog [Organism]**	**Accession ID**	**e-value**	**KO term**^**c**^	**GO term**^**d**^
CC1G_05471T0	0.0	620.5	−9.28				N/A	N/A
CC1G_09700T0	0.0	530.8	−9.05				N/A	N/A
CC1G_03340T0	0.0	374.8	−8.55				N/A	N/A
CC1G_06766T0	0.0	244.6	−7.93				N/A	N/A
CC1G_04060T0	0.0	197.6	−7.63				N/A	N/A
CC1G_06484T0	0.0	147.4	−7.20	hydrophobin [Tricholoma terreum]	AAL05426	9.96E-06	N/A	N/A
CC1G_11781T0	0.0	141.0	−7.14				N/A	N/A
CC1G_04061T0	0.0	104.7	−6.71	hydrophobin [Tricholoma terreum]	AAL05426	9.21E-07	N/A	N/A
CC1G_01315T0	0.0	99.3	−6.63	endochitinase [Amanita muscaria]	CAC35202	2.59E-85	N/A	GO:0004568 GO:0006032
CC1G_12474T0	0.0	83.3	−6.38	xyn11C [Chaetomium thermophilum]	CAD48751	4.69E-68	N/A	GO:0005576 GO:0016798 GO:0045493
CC1G_03570T0	4.5	314.0	−6.11				N/A	N/A
CC1G_14841T0	0.0	66.2	−6.05				N/A	GO:0051015
CC1G_01233T0	0.0	60.9	−5.93	membrane transporter [Cryptococcus neoformans var. neoformans JEC21]	XP_567607	9.43E-37	N/A	N/A
CC1G_10308T0	0.0	57.7	−5.85				N/A	N/A
CC1G_14100T0	0.0	56.6	−5.82	histidine acid phosphatase, putative [Aspergillus clavatus NRRL 1]	XP_001276358	2.98E-06	N/A	N/A

^a^ Relative expression level expressed as tags per 100,000.

^b^ Log_2_ of the expression ratio of Myc/S1 Pri.

^c^ KEGG orthology term.

^d^ Gene Ontology term.

The DEGs were characterized by annotation using the Gene Ontology (GO) database [31]. GO terms were assigned to the DEGs (see “Methods”). A higher proportion of DEGs in the S1-Pri stage could be assigned at least one GO term, with 530 (out of 716, 74%) compared to 146 (out of 324, 45%) as in the Myc stage (Table 3). We have also mapped all the DEGs to the Kyoto Encyclopedia of Genes and Genomes (KEGG) pathways database to investigate molecular interactions and reaction networks [32]. Consistent with our expectations, an overall up-regulation of various cellular processes between the undifferentiated mycelium and the primordium stage 2 days prior to karyogamy was observed. Analysis result from the GO and KEGG is available in the supplemental files 5, 6, 7 and subsections “More DEGs in S1-Pri were assigned a GO term in Gene Ontology analysis” and “KEGG pathways analysis revealed an increased cellular metabolism in S1-Pri” respectively.

### Validations of 5^′^-SAGE with microarray gene expression and real-time RT-PCR

Genome-wide 70-mer oligonucleotide microarray [10,33] experiments were also performed for all predicted genes using similar RNA samples as in the 5^′^-SAGE experiments. The microarray data successfully evaluated expression levels of 8,667 predicted genes (Additional file 4), of which 520 were found to have a false discovery rate <1% using SAM with a 2-fold up-regulation in Myc while 518 were up-regulated in S1-Pri under the same criteria. We randomly selected 18 genes, which covered a spectrum of expression ratios between Myc and S1-Pri genes, for real-time RT-PCR to validate the reliability of the 5^′^-SAGE dataset (Table 6). We found a significant correlation between the real-time PCR results and the 5^′^-SAGE data (R=0.936), suggesting that the 5^′^-SAGE data can reliably measure the expression levels of individual genes. The correlation of the oligonucleotide microarray to 5^′^-SAGE results was also high (R=0.792) for the same set of 18 genes. Thus, both the real-time PCR and microarray data confirmed the expression profiles of the 5^′^-SAGE dataset. However, 562 genes assessed in 5^′^-SAGE were not detected in the microarray data perhaps due to differential sensitivity of sequencing versus hybridization chemistry.

**Table 6:** **Correlation of expression of 18 randomly-selected genes among 5**^**′**^**-SAGE, microarray and real-time RT-PCR**

	**Description**	**5**^**′**^**-SAGE**^**a**^	**Microarray**^**a**^	**Real-time RT-PCR**^**a**^
CC1G_04976	Thiazole biosynthetic enzyme	6.48	3.34	3.80
CC1G_03790	Glutamate decarboxylase	5.55	1.64	3.55
CC1G_00577	CMGC/MAPK protein kinase	4.41	1.41	2.76
CC1G_03377	Protoporphyrinogen oxidase	3.41	−0.17	1.66
CC1G_00689	Thioredoxin	3.16	0.72	2.91
CC1G_07379	Hypothetical protein	1.57	0.19	1.59
CC1G_06934	Hypothetical protein	1.06	−0.85	1.34
CC1G_02958	Actin cytoskeleton protein VIP1	0.82	0.79	0.92
CC1G_10110	Ubiquitin-carboxy protein fusion	−0.05	−0.35	−0.32
CC1G_03278	ATP synthase subunit 5	−0.07	−0.21	−0.41
CC1G_07540	60S ribosomal protein L34-b	−0.16	−0.64	−0.45
CC1G_07277	40S ribosomal protein S14	−0.95	−0.41	−0.78
CC1G_01940	ATP-synthase delta-subunit	−1.34	−0.73	−1.23
CC1G_03707	Prohibitin	−1.43	−0.37	−1.44
CC1G_09336	Basic leucine zipper & W2 domain-containing protein 2	−1.97	−1.17	−1.98
CC1G_06761	Glycine dehydrogenase	−4.42	0.45	−0.82
CC1G_14100	Hypothetical protein	−5.82	−1.53	−3.42
CC1G_01315	Endochitinase	−6.63	−1.45	−4.66
	Correlation of 5^′^-SAGE and real-time PCR: R=0.936			
	Correlation of 5^′^-SAGE and microarray: R=0.792			

^a^ Values expressed as log_2_ ratio of Myc/S1-Pri.

### Gene expression differences among gene families

Among the 324 up-regulated genes in the mycelium stage, only 157 (49%) have a homolog identified through BLASTX search in the NCBI GenBank (e<10^-5^) (Table 3). Compared to 70% (500 out of 716 DEGs) as in the S1-Pri stage, this highlighted that many of the mycelial genes are actually novel. Expression of the hydrophobin 1 gene (*coh1*, CC1G_02183), 3 hydrophobin genes that are in tandem with *coh1* (CC1G_02181, CC1G_02182, CC1G_02184) and 2 other hydrophobin genes (CC1G_04843, CC1G_10189) in Myc revealed that two abundantly expressed ones (CC1G_02183, CC1G_02184) are among the 10 most highly expressed in this stage. All 6 genes show significantly reduced expression in S1-Pri including 4 with no detectable expression. Inverse expression patterns were seen in 4 hydrophobin genes (CC1G_01230, 06484, 04060, 04061), which were highly expressed in S1-Pri but had no detectable transcripts in Myc in 5^′^-SAGE. The expression of the hydrophobin 2 gene (*coh2*, CC1G_02185) was not detected in either developmental stage. These observations are consistent with previous findings in the mushrooms *Lentinula edodes* and *Pleurotus ostreatus* which employ two different sets of hydrophobin genes during fruiting body development [34,35]. These hydrophobins are thought to play alternative structural roles in the respective stages.

A similar phenomenon is observed for the heat shock proteins (HSP). We found 5 HSPs up-regulated in Myc (CC1G_01131, CC1G_02271, CC1G_02341, CC1G_04585, CC1G_15006), whereas 2 are up-regulated in S1-Pri together with 1 heat shock transcription factor (CC1G_04586, CC1G_13982, CC1G_10208). We speculate that the widely employed mycelial cultivation temperature of 37°C compared to the standard fruiting temperature of 25°C [8,10,33] may be partly responsible for this, while other stresses during fruiting development may turn on the corresponding HSPs in the S1-Pri stage [36]. Notably, among the 30 most highly expressed DEGs in Myc, only 7 of them have a detectable homolog in BLASTX search (e<10^-5^). Apart from two of the aforementioned hydrophobin genes, 3 of them (CC1G_07702, CC1G_08572, CC1G_08573) correspond to “mismatched base pair and cruciform DNA recognition protein” which is highly conserved among the fungal kingdom (*A. bisporus* homolog, NCBI accession ID: CAB85690). Little is known of their molecular functions and biological roles in fungal development, but the observations that this family of proteins were among the 5 most highly expressed in Myc and were significantly repressed in S1-Pri highlight their potential involvement in fruiting body development.

### Genes up-regulated in stage 1 primordium

Despite a larger number of genes in the S1-Pri stage for which a homolog can be identified, excluding the mating type genes [37], few other genes have been described as playing a role in fruiting body formation in *C. cinerea*. Some developmentally regulated genes previously characterized include the galectins (β-galactoside binding lectins, CGLs). We showed that the expression of *cgl1* (CC1G_05003), which is highly up-regulated in S1-Pri, is in agreement with a previous report [38]. However, the expression of *cgl2* was not detected in either Myc or S1-Pri stage in our study. One potential explanation for this, as suggested by Boulianne *et al*. [39], is that *cgl2* expression may be repressed by sufficient nutrient levels in the medium (as for Myc) or under light illumination (as for S1-Pri) which resemble the conditions under which our RNA samples were harvested. Our data also revealed the expression of a *cgl3* gene with novel carbohydrate-binding specificities (CC1G_00723) [40], which is highly up-regulated in S1-Pri as well. Cgl3 is dissimilar in protein sequence to both Cgl1 and Cgl2. The most related homolog is a galectin gene from the ectomycorrhizal basidiomycete *Laccaria bicolor* (NCBI accession ID: XP_001877242), yet the homologs are only 58% identical with no other significantly similar sequences identified. The uniqueness and differential expression of *cgl3* probably deserves a deeper investigation.

The expression of tubulin genes has been previously explored [41-43]. Examination of this gene family found three components of microtubules: alpha1 tubulin (CC1G_01375), alpha2 tubulin (CC1G_00146), beta tubulin (CC1G_04743), which are all up-regulated in S1-Pri with a similar 5-fold of change, accompanied by a 3-fold increase of a tubulin-specific chaperone (CC1G_00477) suggesting that microtubule formation is systematically regulated and necessary for early fruiting events.

### More DEGs in S1-Pri were assigned a GO term in Gene Ontology analysis

**Figure 3:**
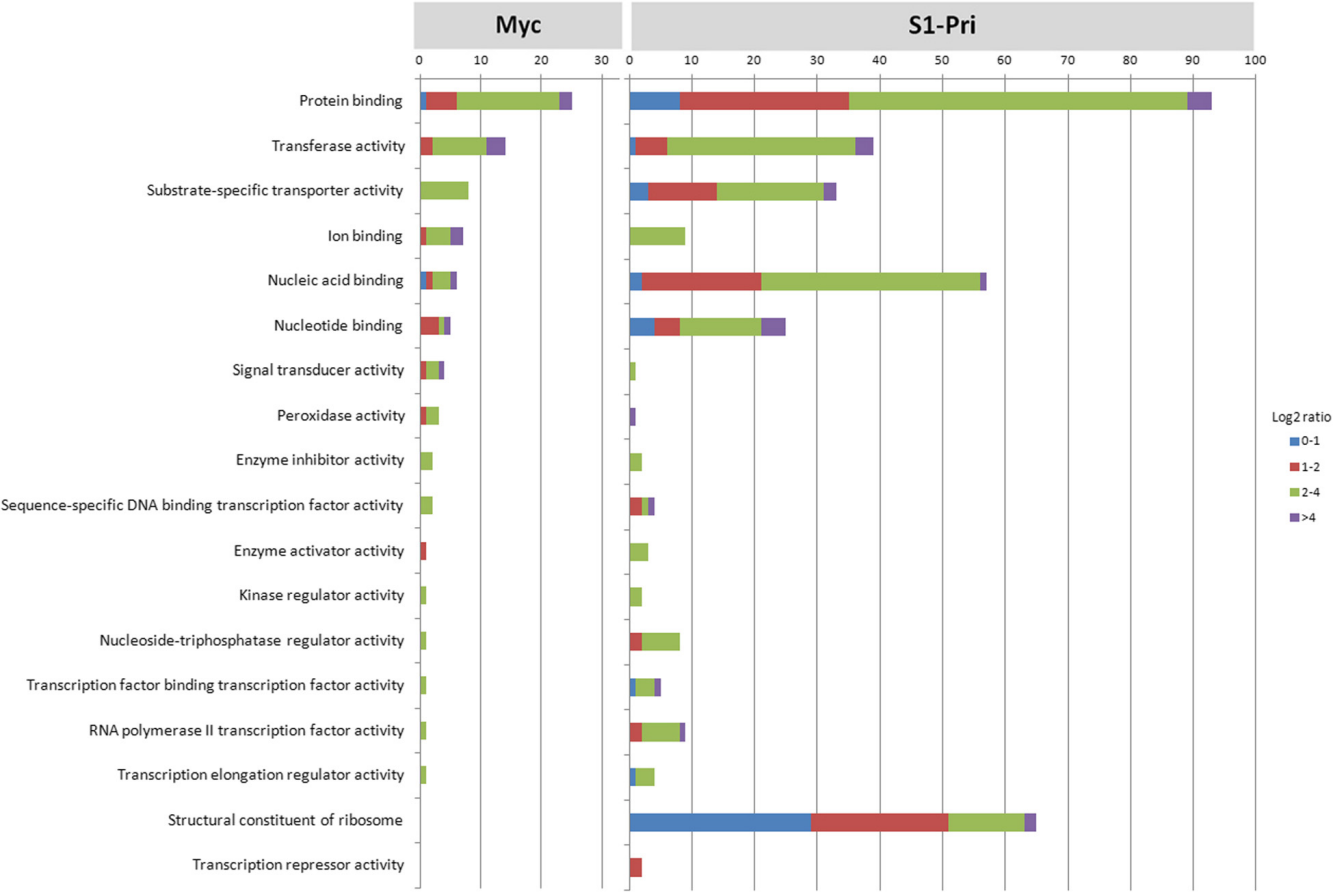
**Assignment of DEGs to 18 selected Molecular Function in Gene Ontology.** Both Myc and S1-Pri DEGs were assigned to Gene Ontology and 18 selected Molecular Functions are presented to show that gene sets involved in many important cellular activities such as transcription factor binding, DNA/RNA/protein binding, enzyme regulation, transcriptional activation/repression and translation had to be suppressed and induced simultaneously during fruiting body development.

We inferred the gene characteristics of the Myc and S1-Pri DEGs using the Gene Ontology (GO) [31] database. A total of 146 Myc DEGs were assigned to GO terms consisting of 92 cellular components, 111 molecular functions and 99 biological processes, while 530 S1-Pri DEGs were assigned to 423 cellular components, 411 molecular functions and 409 biological processes respectively (Additional file 5, we recommend viewing using Gene Ontology Browsing Utility [44]). We specifically focused on those assigned to the category “Molecular function” and “Biological Process” for further investigation. More DEGs in the S1-Pri stage than Myc can be assigned a role in molecular function (34% in Myc against 56% in S1-Pri) and biological process (31% in Myc against 57% in S1-Pri), again highlighting the phenomenon that many mycelial genes are novel. We observed a large number of S1-Pri DEGs being assigned to molecular functions including transcription factor binding, DNA/RNA binding, GTPase regulator activity, transcriptional activator/repressor activity and ion transport activity (Figure 3). Concerning gene expression, we identified transcription regulators (5 in Myc; 42 in S1-Pri), which implies that genes are under different transcriptional regulation during fruiting body formation.

**Figure 4:**
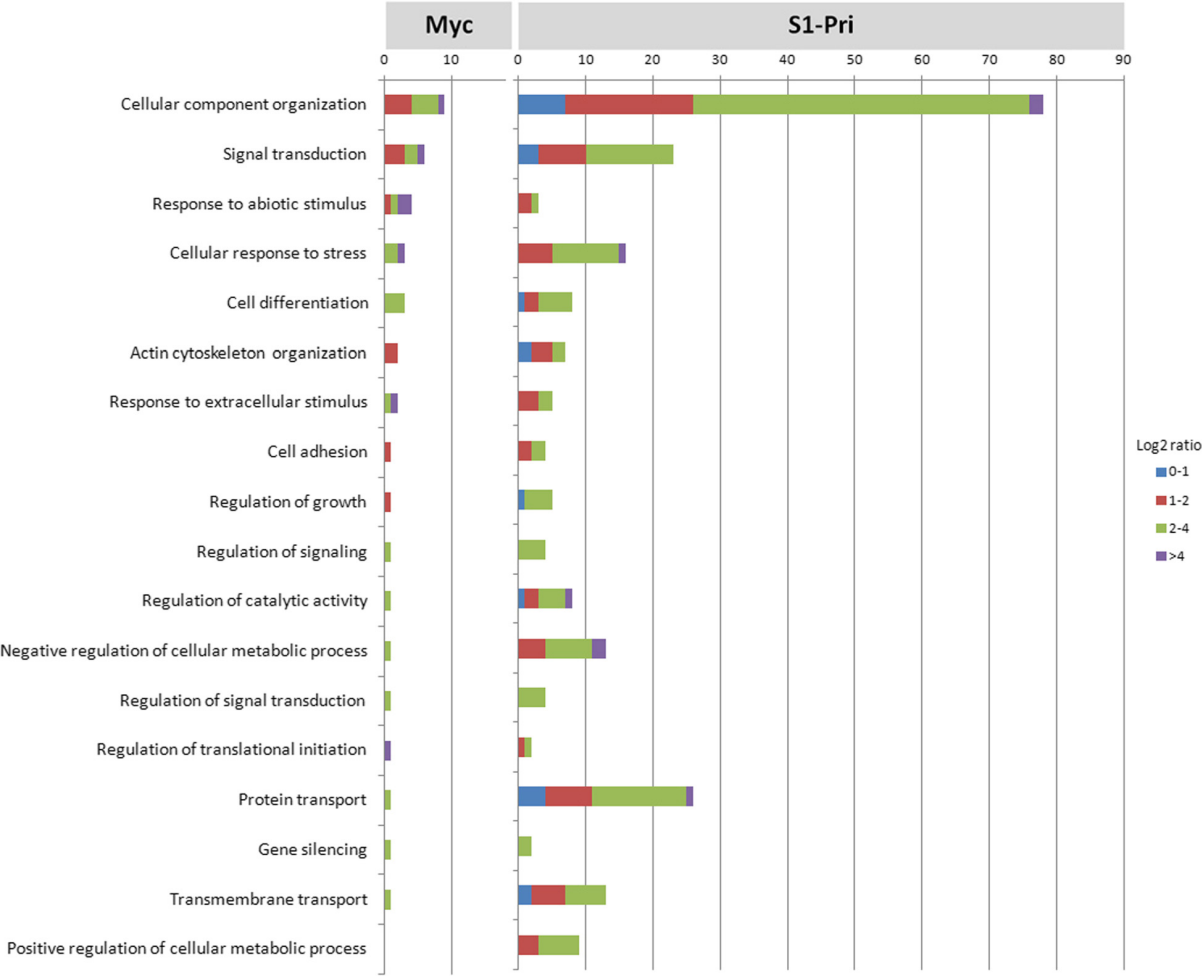
**Assignment of DEGs to 18 selected Biological Process in Gene Ontology.** Eighteen selected Biological Processes in Gene Ontology are presented to show that many biological processes such as cellular organization, signal transduction, environmental sensing, stress response, cellular process regulation and protein transport are significantly up-regulated in S1-Pri compared to Myc during fruiting body formation.

The biological processes observed among the DEGs could play important roles during fruiting body formation including cell adhesion, signal transduction, sensing of extracellular stimuli, responding to stress and in the regulation of cellular process (Figure 4). Notably, in addition to the components of the cAMP and MAP kinase signaling pathways to be discussed below, we uncovered 31 DEGs involved in other signal transduction processes (6 in Myc; 20 in S1-Pri) and regulation of signal transduction (1 in Myc; 4 in S1-Pri) through GO analysis. This suggests different signaling cascades may be required to transduce various signals into either mycelial growth or fruiting body development [45,46]. Many of the Myc DEGs cannot be assigned to any GO term, but despite this a similar number of genes were assigned to “response to abiotic stimulus” and “response to nutrient levels” when compared to the S1-Pri DEGs, suggesting that *C. cinerea* may employ a different set of genes to sense the environment and to transduce the corresponding signals to development. Two of the terms are even unique to the Myc stage, which include “a specific receptor and receptor signaling protein functions” and “certain antioxidant activities”.

### Involvement of nutrient-related pathways in fruiting body development

**Figure 5:**
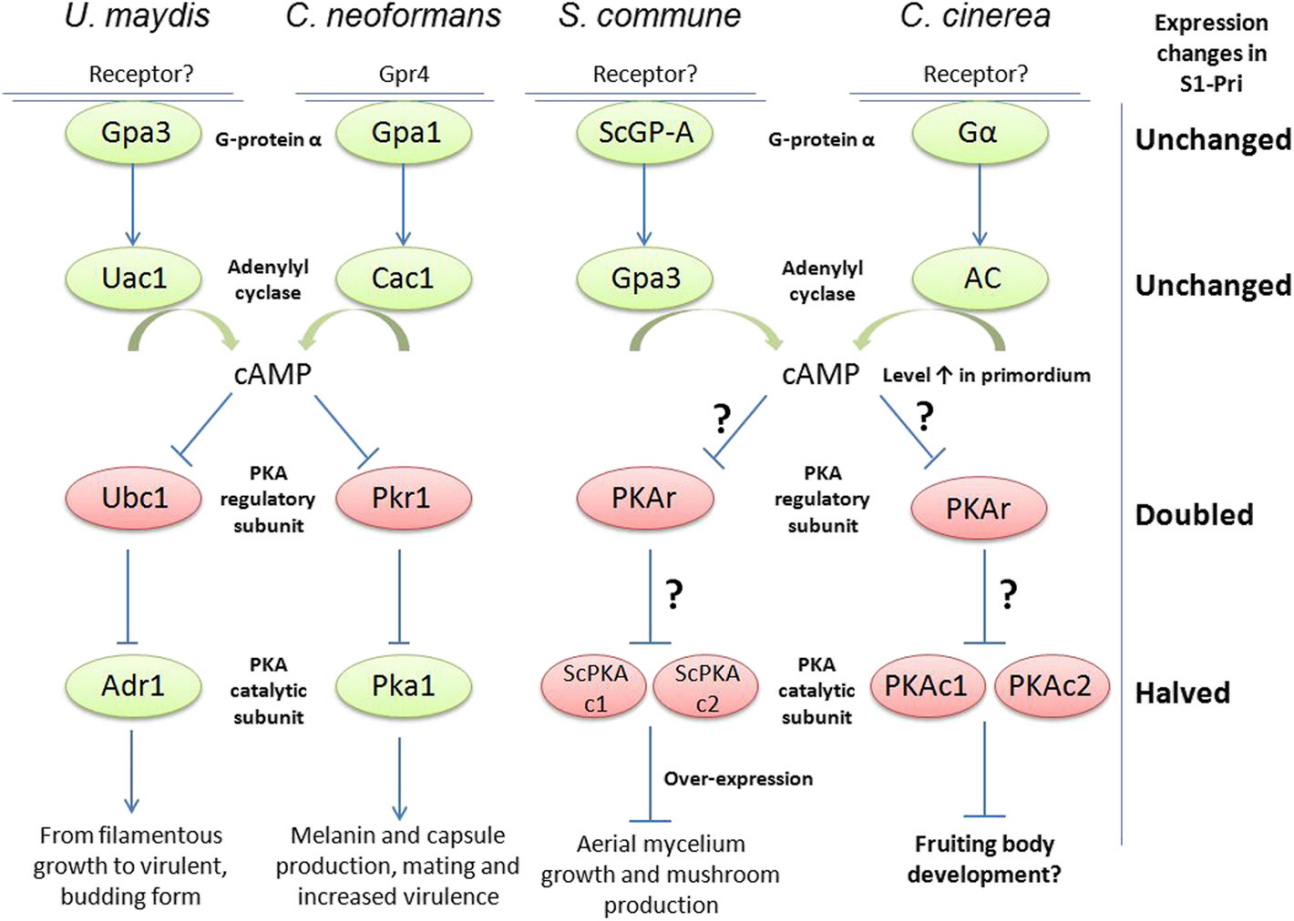
**The cAMP signaling pathway and morphogenesis in four selected basidiomycetes.** The cAMP signaling pathway in four basidiomycetes *Ustilago maydis*, *Cryptococcus neoformans*, *Schizophyllum commune* and *C. cinerea* (adapted from Lengeler et al., 2000 and Palmer and Horton, 2006). The G-protein-coupled receptor had only been identified in *C. neoformans*. G-protein α: “cAMP-type” alpha subunit of the heterotrimeric G-protein. PKA: protein kinase A. Proteins with activating functions are colored in green, and proteins with known/putative repressive functions are colored in red.

In addition to the 2 aforementioned genes encoding galectins *cgl1* and *cgl3*, which were assigned directly to the category ‘sporocarp (fruiting body) development’ [39,40], 2 Myc and 4 S1-Pri genes were predicted to play a role in response to nutrient levels, as nutrient depletion is believed to be one of the primary factors for fruiting [2,8]. Three of these genes (CC1G_00577, CC1G_00269 and CC1G_11556) are highly conserved in animals and fungi but not in plants, and the remaining three (CC1G_03709, CC1G_04470 and CC1G_12113) are highly conserved in fungi. These six proteins consist of one protein kinase, one rheb GTPase, one endopeptidase and three hypothetical proteins. Nevertheless, they are not assigned to any known nutrient-related pathways including the cAMP and MAP kinase pathways discussed below. Meanwhile, we specifically investigated the cAMP signaling pathway, which functions in parallel with the MAPK cascade. This pathway involves a novel G protein-coupled receptor, G protein alpha, adenylyl cyclase, cyclic AMP (cAMP), cAMP-dependent protein kinase and Ras [45]. The homolog of *Cryptococcus neoformans* Gα protein Gpa1 and *U. maydis* Gpa3, a conserved subgroup of fungal “cAMP-type” Gα proteins, has been implicated to respond primarily to nutrient deprivation signals by inducing cAMP levels and subsequently activating cAMP-dependent protein kinase (PKA) [47]. Physiological studies revealed that “cAMP-type” Gα protein homolog and adenylyl cyclase controlled cAMP production in response to glucose, and that intracellular cAMP levels rises in *C. cinerea* during hyphal knot and primordium formation as a result of Gα protein activation [45,48]. Since our 5^′^-SAGE showed reduced expression for many of the components in the cAMP pathway, we employed real-time PCR to verify their expression levels. We demonstrated that the expression of both the *C. cinerea gpa1* homolog (CC1G_09275) and the adenylyl cyclase gene (CC1G_02340) remain approximately unchanged between the Myc and the S1-Pri stage. However, a 1.9-fold up-regulation of the cAMP-dependent protein kinase (PKA) regulatory subunit (yeast Bcy1 homolog, CC1G_00440) [49] was recorded, and expression of two genes encoding the cAMP-dependent protein kinase catalytic subunit (CC1G_05183 & 04466) was reduced by half. This is somewhat contradictory to the observation by Swamy *et al*. [50], as they reported that adenylyl cyclase level was decreasing in mated dikaryon after it peaked at 6 days post-incubation. It is to be noted that they did not compare PKA activity between the mycelium and the primordium stages. However, in line with the observation by Yamagishi *et al*. [51], expression of PKA was also greatly reduced in mushroom-forming *Schizophyllum commune* dikaryons. This intriguing observation of an apparently down-regulation of the known downstream targets of a Gα protein-mediated signaling pathway argues against the role of PKA as a downstream effector controlling mushroom formation. We hypothesize that the down-regulation of PKA may be responsible during fruiting body formation for the suppression of its targets, likely a variety of transcription factors, and that the observed increase in cAMP levels may serve roles other than activating PKA as in *C. neoformans* and *U. maydis* (Figure 5).

### Involvement of the MAP kinase pathway in fruiting body development

As far as the cAMP-PKA signaling pathway is concerned, we are also interested in investigating the MAP kinase (MAPK) signaling pathway, since it has been well-documented that the two pathways function in parallel with cross talk between them [46,52,53]. In *U. maydis*, the MAP kinase homolog Ubc3 is essential for pheromone responses, mating and filamentation [54], and similar developmental roles have also been described in budding yeast [55], *C. neoformans*[56] and *Neurospora crassa*[57]. However, the role of the MAPK cascade in fruiting body development in basidiomycetes has not been studied in great detail save for observations such as MAPK was found preferentially expressed in primordium and young fruiting bodies in *L. edodes*[58]. It was also shown that mutation of the protein kinase regulator *Cc.ubc2* (CC1G_00975) gene blocked phosphorylation of a presumptive MAP kinase [59]. Nevertheless, not only was expression of the *Cc.ubc2* gene not detected in either developmental stage in our 5^′^-SAGE data, we also showed low expression levels for the components in the MAP kinase pathway. However, real-time PCR demonstrated that two loci of MAP kinase (CC1G_07620 and CC1G_03633) were up-regulated by 0.4 fold and down-regulated by 70% in S1-Pri respectively. Consistent with such results, expression of MAP kinase kinase loci (MAPKK) (CC1G_03368 and CC1G_02205) were up-regulated by 1.2-fold and down-regulated by 78% in S1-Pri respectively. It is unclear whether the two apparent subsets of MAPK/MAPKK combinations function independently, despite the high similarity in the conserved functional domains in these kinases. Accordingly, the budding yeast Ras2 homolog (CC1G_04430), which has been implicated to function as an upstream activator of both the cAMP-PKA and MAPK signaling cascades [60,61], was also found to be moderately up-regulated by 0.5-fold in S1-Pri. This suggests that the MAPK pathway and its activator may still be necessary although not up-regulated, for fruiting body development in *C. cinerea*.

### KEGG pathways analysis revealed an increased cellular metabolism in S1-Pri

Among the 270 up-regulated genes (38%) in S1-Pri in which a KEGG ortholog could be identified (Table 3), 62 (23%) were annotated to be constituents of ribosome and most of the remaining were mapped to metabolic pathways involving oxidative phosphorylation (27), nucleotide metabolism (14), TCA cycle (6), sugar metabolism (7), amino acids metabolism (20) and protein degradation (12) (Additional file 6). This implies a significant higher demand for energy production, DNA synthesis, protein synthesis machinery and turnover during fruiting body development. In contrast, only 56 up-regulated genes (17%) in Myc were assigned a KEGG ortholog, and almost all of them were mapped to basic cellular metabolism pathways (Additional file 7). Furthermore, these 56 genes were assigned to as many as 69 different pathways and most of which only had one and two mapped components (47 and 19 respectively), thereby implying that few pathways are preferentially up-regulated during mycelial growth. Moreover, a remarkable 20% of Myc and 45% of S1-Pri DEGs with KEGG ortholog could not be assigned to a KEGG pathway.

### Novel pathways potentially involved in fruiting body development

**Figure 6:**
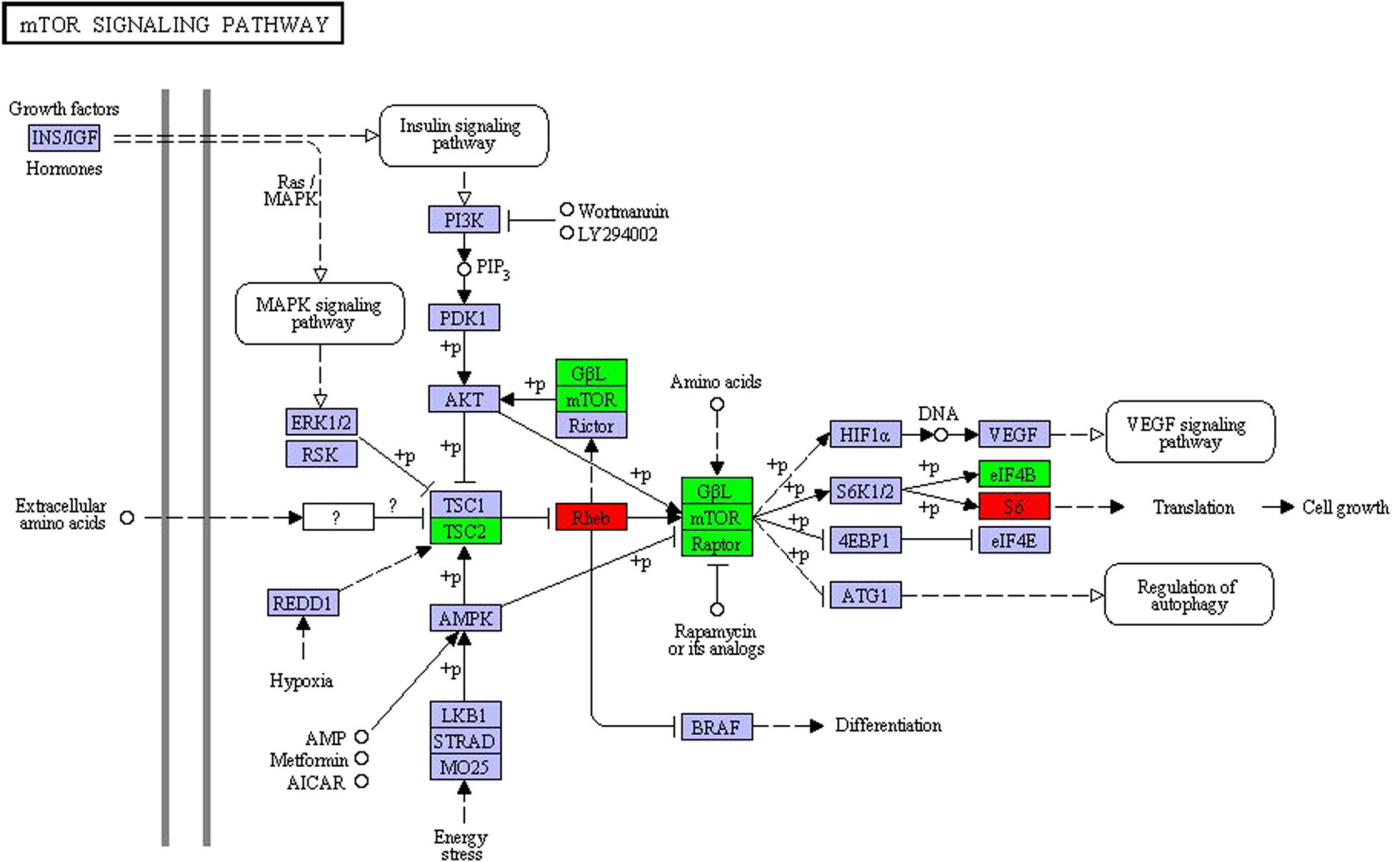
**The mTOR signaling pathway in KEGG pathway analysis.** The mTOR signaling pathway is among the S1-Pri-up-regulated pathways in KEGG analysis. Rheb and ribosomal protein small subunit s6e (red boxes) were identified through 5^′^-SAGE as DEGs in the S1-Pri stage. Expression of five other components (green boxes) were verified by real-time PCR.

Among the up-regulated pathways in S1-Pri, we are particularly interested in novel signaling pathways [62]. Two up-regulated genes in S1-Pri were assigned to the mTOR signaling pathways, and real time PCR confirmed the 5^′^-SAGE observations for these two DEGs (CC1G_10718 & CC1G_00269). The mTOR signaling pathway is of particular interest as the TOR pathway is well-conserved from animals to fungi [63-65] (Figure 6). TOR (Target of Rapamycin) is a serine/threonine protein kinase controlling a wide range of cellular events in response to different environmental cues such as stimulation by growth factors, changes in nutrient conditions and fluctuation in energy, and is specifically inhibited by FKBP12 protein together with rapamycin [66]. During amino acid (nitrogen) starvation, FKBP12 homologs interfere with TOR and thereby inhibit cell growth. We showed that although TOR (CC1G_00894) and its associated protein Raptor (CC1G_00662) had similar expression between Myc and S1-Pri stages, our 5^′^-SAGE data and real-time PCR recorded a 8.5-fold and 10.6 fold up-regulation of a Rheb homolog (CC1G_00269), a Ras-like small GTPase, which is able to prevent FKBP12 inhibition of TOR in a GTP-dependent manner such that TOR is activated in turn [66,67]. In addition, although the TSC1 homolog cannot be identified, expression of the TSC2 component (CC1G_15321) of the critical TOR negative regulator TSC1-TSC2 (tuberous sclerosis complex) complex remained unchanged. We also demonstrated that two end effectors of the mTOR pathway, translation initiation factor 4B (CC1G_10919) and ribosomal protein small subunit s6e (CC1G_10718), showed a concurrent 1.6-fold up-regulation. We also observed a 2.6-fold up-regulation of a G-protein beta-like homolog (CC1G_06967) that is implicated to play a role in nutrient sensing. Taken together, our observations suggest that this Rheb GTPase homolog and the TOR signaling pathway functions as one of the essential regulators during fruiting body formation.

Furthermore, the protein riboflavin-aldehyde forming (Raf) enzyme was found to be differentially expressed in primordium in both *L. edodes*[34] and *Agaricus bisporus*[68] during fruiting body formation. Although our 5^′^-SAGE data only showed a slight increase in the expression of the *raf* gene, 4 DEGs (CC1G_13718, 01137, 04209, 11697) in the S1-Pri stage were mapped to the pathway riboflavin metabolism. Genes CC1G_13718 and CC1G_01137 encode the alpha and beta chain of riboflavin synthase, suggesting that an up-regulation of riboflavin synthesis may be a necessary complement to the increase in expression of the Raf enzyme in *C. cinerea* as well as other fruiting body-forming basidiomycetes.

### Protein domain enrichment

We searched all the annotated ORFs for protein domains using NCBI Conserved Domains [69], NCBI Protein Clusters [70], Pfam [71] and COG [72] databases. A total of 5,868 conserved domains were assigned to 3,751 ORFs at an e-value threshold of e<10^-20^. The occurrence of conserved domains in the Myc and S1-Pri DEGs were compared against all the ORFs. A domain is defined as ‘enriched’ in the set of DEGs if it has a *p*-value lower than 0.05 in the Fisher’s Exact Test. We identified 118 enriched domains in the Myc stage (Additional file 8) and 115 in the S1-Pri stage (Table 3) (Additional file 9). Intriguingly, as many as 93 Myc DEGs-enriched domains are present only once across the genome, and they are all represented in the Myc DEGs but never in the S1-Pri DEGs. This suggests types of protein functions suppressed during fruiting body formation.

We observed an enrichment of PIWI domains [73] in the Myc DEGs. Since this family plays a role in RNA silencing, as found in piwi proteins and a large number of piwi-related nucleic acid-binding proteins, our observations may provide some insights into gene regulation as fruiting occurs [74]. Among these 115 S1-Pri DEGs-enriched domains, 42 belong to the Ras/Rab/Ran families and 11 to the ribosomal protein domains, which are consistent with previous observations of a significant up-regulation of signal transduction and protein synthesis events. Other highly enriched domains in S1-Pri DEGs include 10 enoyl-CoA hydratase, 8 tubulin, 7 ARF (ADP ribosylation factor)/ARF-like domains found in GTP-binding proteins of the Ras superfamily [75], which deserve further investigation to uncover their roles during fruiting body development.

### Genes unique to fruiting body-forming basidiomycetes and *C. cinerea*

We searched all differentially expressed genes in both Myc and S1-Pri stages against the NCBI BLAST database and specifically against our in-house sequence database which consisted of the protein sequences of *Saccharomyces cerevisiae*, filamentous fungi (*C. neoformans*, *U. maydis*, *Aspergillus fumigatus*), *L. edodes*[76], and a number of fruiting body-forming basidiomycetes including *L. bicolor*[77], *Phanerochaete chrysosporium*[78], *Postia placenta*[79], *S. commune*[80], and two others *P. ostreatus* and *A. bisporus* obtained from the JGI Genome Portal [81]. In order to identify the DEGs that are unique to the fruiting body-forming basidiomycetes, we filtered out the genes in the blast reports that were dissimilar in sequences (e>10^-2^) and used a threshold of e<10^-10^ to indicate sequence homology among the seven basidiomycetous species. Under these criteria, we identified 22 Myc and 30 S1-Pri DEGs that are uniquely found in at least two fruiting body-forming basidiomycetes (Additional file 10). We speculate that these genes are fundamental and unique to fruiting body development in basidiomycetes despite their roles being unclear. Intriguingly, using the same criterion of e<10^-10^, we identified 41 Myc and 22 S1-Pri DEGs that were found only in the *C. cinerea* genome (Additional file 11). These genes do not bear any conserved protein domains and were not assigned to any GO terms or KEGG pathways. They may be novel and specific to fruiting body development in *C. cinerea*.

## Conclusions

We have demonstrated the capability of a combined approach of the 5^′^-SAGE procedures and high-throughput pyrosequencing to evaluate the transcriptomes of *C. cinerea* at the mycelium and stage 1 primoridum stages respectively. Expression of more than 3,000 genes were assessed, and we showed that one-third of these genes were preferentially expressed in either growth stage, suggesting an overall transcriptomic switch during the transition from mycelium to primordium. Two different sets of structural and functional proteins were found to be uniquely employed in either developmental stage. We demonstrated that sensing the nutrient levels and the environment and response to these changes, in addition to an up-regulation of cellular processes including energy production, DNA/protein synthesis, protein degradation, are crucial for fruiting body development. We also hypothesize an independent regulation of the MAPK and cAMP signaling pathways and an up-regulation of the TOR signaling pathway as necessary requirements. A wealth of candidate genes and anti-sense transcripts potentially related to early fruiting events were also uncovered. The 5^′^-SAGE data presented in this study serves to advance our understanding of the molecular mechanisms of fruiting body development in *C. cinerea*, which may as well be applicable to the other basidiomycetes that form fruiting bodies.

## Methods

### Strains and culturing

A dikaryotic *C. cinerea* strain, mated from two wild-type monokaryon strains J6;5–5 and J6;5–4 [82], was used in this study. The two monokaryotic strains were re-generated from single spore isolates of a dikaryotic strain which had been backcrossed with the reference strain Okayama 7 #130, for which its genome sequence is available, for 5 generations. Mycelium (Myc) was grown at 37°C in darkness on solid YMG medium and harvested when the mycelium just fully covered the Petri dish (approx. 7 days). Stage 1 primordium (S1-Pri) was grown at 25°C under a light/dark regime of 14/10 hr [10,33,82] and harvested by picking individual primordia from the base when they were at 2 days prior to karyogamy (height of about 5 mm).

### Ditag formation

Total RNA was extracted by TRI® reagent (Molecular Research Center, Inc) and Poly(A)^+^ mRNA was furthered isolated using the PolyATract® mRNA isolation system (Promega). First strand cDNA was synthesized using SuperScript™ III First-Strand Synthesis System for qRT-PCR (Invitrogen). Two different template switching oligos were employed for each developmental stage. Reverse transcription was performed at 42°C for 90 minutes. Second strand cDNA was then synthesized by low cycle primer extension using Advantage® 2 polymerase (Clontech). Double-stranded cDNA was purified by QIAquick PCR purification kit (Qiagen) and checked by electrophoresis on a 1.5% agarose gel.

The cDNAs were digested by 10units of *Mme*I (New England Biolabs) at 37°C for 2 hours. The digested products were extracted by phenol-chloroform and precipitated by ammonium acetate and ethanol at −20°C overnight, then re-suspended at low salt TE (2.5 mM Tris HCl, 0.25 mM EDTA, pH8.0) and electrophoresed on 15% polyacrylamide gel (Bio-Rad). After electrophoresis, the ~50 bp bands were excised, disrupted and the DNA contents were eluted and pooled. Following ethanol precipitation with ammonium acetate at −70°C for 4 h, pellets were collected and resuspended in low-salt TE buffer.

The pools of *Mme*I-digested fragments were ligated by 600units of T4 DNA ligase at 16°C overnight to form 100 bp ditags. The ligation mixtures were divided into half and PCR-amplified. The two PCR reactions were then pooled together, extracted by phenol/chloroform and precipitated with ammonium acetate and ethanol. Pellets were collected by centrifugation and resuspended in 15 μl ultra-pure water.

### High-throughput pyrosequencing

Prior to pyrosequencing, the identity of the 100 bp ditags were checked by 2% agarose gel electrophoresis and TA cloning using cloning vector pMD-18 T (Takara Biotechnology). Products of PCR screening with expected size were purified and sequenced for confirmation of the identity of the ditags. The mycelial and primordial ditags were sequenced with a GS20 sequencer in two separate metrics at 454 Life Sciences (Connecticut, U.S.A.).

### Tag extraction and genome mapping

The accuracy of all sequence reads were checked using a Phred-equivalent quality score. Perl scripts were written to remove low-quality sequences, extract valid ditags and subsequently extract individual tags. All tags were mapped to the *C. cinerea* genome sequence obtained from the Broad Institute using Perl scripts [30]. Only tags with a single exact match were retained for further analysis. Singleton tags were also discarded.

### Identification of differentially expressed genes

The expression level of individual genes was assessed by summing up the occurrence of all the tags mapped to the putative 5^′^-UTR and dividing this number by the total occurrence of all genome-mapped tags, then multiplying by 100,000, following which they were expressed as tags per hundred thousand in the corresponding stage. Differentially expressed genes (DEGs) between the Myc and S1-Pri stages were defined by a *p*-value of 0.05 in the pairwise Fisher Exact Test.

### GO and KEGG analysis

Protein homologs for all *C. cinerea* predicted gene models were assigned according to the best hit in BLASTX [83] against the NCBI non-redundant protein sequence database (e<10^-5^). Gene Ontology (GO) terms were assigned to protein homologs through BLAST2GO (version 2.4.2) using default parameters [31]. In subsequent GO term analysis of the differential expression data, corresponding level 2 parent terms were used in “biological process”, “molecular function” and “cellular component”. KEGG orthologs (KO) and KEGG biological pathways were also assigned through the KEGG Automatic Annotation Server (KAAS) [32] using the following parameters: BBH (bi-directional best hit method), and all fungal species as the representative gene set.

### Oligonucleotide microarray

The microarray platform, hybridization methods and data analysis have been described [10,33]. More than 13,000 oligonucleotides were designed using ArrayOligoSelector and printed randomly in duplicates on the slides. They were designed to minimize chances of a secondary match, and to be 3^′^ biased where possible. Myc and S1-Pri RNA samples from biological replicates were isolated from stages mentioned in “Strains and culturing”. First-strand cDNA was synthesized and labeled using Superscript Indirect cDNA Labeling System (Invitrogen) with alexa-fluor 635 and 532. Arrays were hybridized in four replicates. Data were captured and analyzed with GenePix 4200A scanner and GenePix Pro software (Molecular Devices). Data for a given oligonucleotide were included if two or more of the four replicates contained data for both probes. Intra-slide normalization and log_2_ transformation were performed using OLIN (Optimized local intensity-dependent normalization) and data were Z-adjusted to facilitate comparisons among arrays. Significance analysis of microarrays (SAM) was used to determine significant differences in gene expression between samples and the control.

### Quantitative real-time RT-PCR

The reliability of 5^′^-SAGE data was verified by examining expression of genes showing higher expression in Myc or S1-Pri and similar expression levels. Total RNAs were isolated from Myc and S1-Pri stages mentioned in “Strains and culturing” and were DNase-treated prior to first-strand cDNA synthesis using TaqMan® Reverse Transcription (RT) reagents (Applied Biosystems). RT-PCR primers were designed using the OLIGO™ software (version 4.0) (National Biosciences). Real-time RT-PCR was performed on the MiniOpticon™ Real-Time PCR detection system (Bio-Rad) using iQ™ SYBR® Green supermix (Bio-Rad) according to the manufacturer’s instructions. Reaction volumes of 20 μl containing 2 μl of 10X diluted cDNA were used and samples were prepared from biological replicates for each developmental stage and primer pair. Gene CC1G_03085 encoding a clathrin coat assembly protein was selected as the control for normalization. Melting curve analysis was also performed according to the manufacturer’s instructions (Additional file 12 and Additional file 13).

### Availability of supporting data

The data sets supporting the results of this article are available in the NCBI Gene Expression Omnibus (GEO) repository under accession GSE40722.

## Abbreviations

5'-SAGE: 5'-Serial Analysis of Gene Expression; Myc: Mycelium; S1-Pri: Stage 1 primordium; DEG: Differentially expressed gene; TSS: Transcription start site; UTR: Untranslated region; GO: Gene Ontology; KEGG: Kyoto Encyclopedia of Genes and Genomes; RT-PCR: Reverse transcription PCR; HSP: Heat shock protein; cAMP: cyclic adenosine monophosphate; MAPK: Mitogen-activated protein kinase; MAPKK: mitogen-activated protein kinase kinase; PKA: Protein kinase A; TOR: Target of rapamycin.

## Competing interests

The authors declare that they have no competing interests.

## Authors’ contributions

Listing those who: Conceived and designed the experiments: HSK, PJP & MEZ. Performed the experiments: CKC & SKW. Analyzed sequence data: CHA, CKC and JES. Drafted the manuscript: CKC. Revised the manuscript: HSK, JES, & PJP. All authors read and approved the final manuscript.

## Supplementary Material

Additional file 1 **List of 3270 genes of which expression is detected in either Myc or S1-Pri stage.** Expression level is evaluated by summing up the counts of all tags mapped to the putative 5^′^-UTR, divided by the total tag counts in the respective stage, and expressed in tags per 100,000. Click here for file

Additional file 2 **List of 324 genes differentially expressed in the Myc stage.** Expression level is evaluated by summing up the counts of all tags mapped to the putative 5^′^-UTR, divided by the total tag counts in the Myc stage, and expressed in tags per 100,000. Differential expression is defined by p<0.05 in Fisher’s exact test. Click here for file

Additional file 3 **List of 716 genes differentially expressed in the S1-Pri stage.** Expression level is evaluated by summing up the counts of all tags mapped to the putative 5^′^-UTR, divided by the total tag counts in the S1-Pri stage, and expressed in tags per 100,000. Differential expression is defined by p<0.05 in Fisher’s exact test. Click here for file

Additional file 4 **Microarray data for the 8667 genes after quality filtering.** M_006_z to M_009_z represent four replicate experiments. Values are given as log2 ratio of expression level of Myc divided by that of S1-Pri. Click here for file

Additional file 5 **Gene ontology terms assigned to the differentially expressed genes in both Myc and S1-Pri.** We recommend viewing using Gene Ontology Browsing Utility, available at //gobu.iis.sinica.edu.tw. Run “gobuCmd.bat” in the “gobu” folder after extraction of the downloaded .zip file, wait for the program interface to pop up and load the .txt file into the program. Click here for file

Additional file 6 **KEGG pathway maps assigned to S1-Pri DEGs.** KEGG Orthology (KO) id and description can be found in the last two columns of additional file 2. Click here for file

Additional file 7 **KEGG pathway maps assigned to Myc DEGs.** KEGG Orthology (KO) id and description can be found in the last two columns of additional file 1. Click here for file

Additional file 8 Protein domains enriched in the Myc DEGs. Click here for file

Additional file 9 Protein domains enriched in the S1-Pri DEGs. Click here for file

Additional file 10 **Myc and S1-Pri DEGs that are unique to at least two fruiting body-forming basidiomycetes.** Ab: *Agaricus bisporus*; Le: *Lentinula edodes*; Lb: *Laccaria bicolor*; Po: *Pleurotus ostreatus*; Sc: *Schizophyllum commune*; Pc: *Phanerochaete chrysosporium*; Pp: *Postia placenta*. Click here for file

Additional file 11 **Myc and S1-Pri DEGs that are unique to *C. cinerea.*** Click here for file

Additional file 12 **Primers used in real-time RT-PCR for cross-validation of 5**^**′**^**-SAGE and microarray.** Click here for file

Additional file 13 Primers used in real-time RT-PCR for expression validation of additional selected genes. Click here for file
